# Deformation-induced trace element redistribution in zircon revealed using atom probe tomography

**DOI:** 10.1038/ncomms10490

**Published:** 2016-02-12

**Authors:** Sandra Piazolo, Alexandre La Fontaine, Patrick Trimby, Simon Harley, Limei Yang, Richard Armstrong, Julie M. Cairney

**Affiliations:** 1 Australian Research Council Centre of Excellence for Core to Crust Fluid Systems/GEMOC, Department of Earth and Planetary Sciences, Macquarie University, Sydney, New South Wales 2109, Australia; 2 Australian Centre for Microscopy and Microanalysis, University of Sydney, Sydney, New South Wales 2006, Australia; 3 School of Geosciences, Grant Institute, University of Edinburgh, Edinburgh EH9 3JW, UK; 4 Research School of Earth Sciences, Australian National University, Canberra, Australian Capital Territory 0200, Australia

## Abstract

Trace elements diffuse negligible distances through the pristine crystal lattice in minerals: this is a fundamental assumption when using them to decipher geological processes. For example, the reliable use of the mineral zircon (ZrSiO_4_) as a U-Th-Pb geochronometer and trace element monitor requires minimal radiogenic isotope and trace element mobility. Here, using atom probe tomography, we document the effects of crystal–plastic deformation on atomic-scale elemental distributions in zircon revealing sub-micrometre-scale mechanisms of trace element mobility. Dislocations that move through the lattice accumulate U and other trace elements. Pipe diffusion along dislocation arrays connected to a chemical or structural sink results in continuous removal of selected elements (for example, Pb), even after deformation has ceased. However, in disconnected dislocations, trace elements remain locked. Our findings have important implications for the use of zircon as a geochronometer, and highlight the importance of deformation on trace element redistribution in minerals and engineering materials.

## Abstract (web summary)

Pb/U ratios and trace element distributions within zircon grains are used to determine a rock's age and geological processes, but the degree of element immobility and diffusion is uncertain. Here, the authors use atom probe tomography to reveal sub-micron scale mechanisms of trace element mobility.

## Graphical abstract

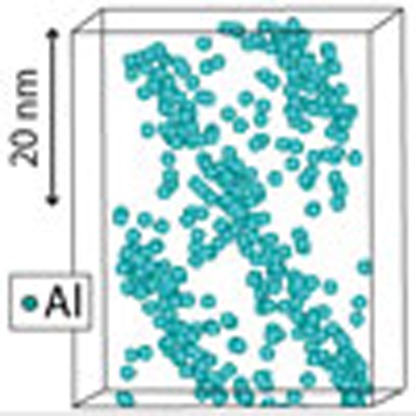

The recent development of a range of high-resolution, chemically sensitive analytical techniques has enabled routine geochemical characterization of geological samples; this allows the use of trace element distributions in single grains as indicators of the Earth's large-scale processes^1^,^2^. Fundamental to these studies is the assumption of element immobility and/or predictable element diffusion. Theoretically, deformation may induce differential element mobility^3^ resulting in substantial changes to both absolute and relative element concentrations. However, even though many rocks undergo crystal–plastic deformation, the link between trace element mobility and deformation is still poorly understood.

Zircon is one of the main minerals routinely analysed *in situ* for several trace elements. The ability of the zircon lattice to incorporate U, Th, Hf and a number of geochemically important rare earth elements, while remaining physically and chemically robust^4^, has resulted in the use of zircon geochemistry in many geoscience disciplines (igneous petrology^5^; provenance studies^6^; metamorphic processes^4^,^7^; Earth evolution^2^,^8^,^9^). Although experimental determination of diffusion rates within pristine zircons shows that substantial Pb diffusion should only occur at extreme temperatures^10^, there is some evidence that Pb diffusion can take place at lower temperatures^11^. This is often attributed to the annealing of regions of radiation damage within the crystalline lattice^12^,^13^. Such damaged (metamict) domains are only partially crystalline, may be porous^12^,^14^, and are usually cited as the cause of either relative Pb-loss (discordance) or Pb-gain (reverse discordance) recorded on the micrometre scale^13^,^14^,^15^. Recent studies show diffusion only over small distances (<100 nm) resulting in Pb clusters^6^ and some micrometre-scale redistribution^16^. Although measurable changes in trace element concentrations have been linked to micrometre-scale deformation structures^17^,^18^,^19^, atomic-scale evidence of the relevant processes is lacking. Consequently, it is not yet possible to predict how these changes may influence the accuracy of dating or our ability to utilize trace element data.

In metals and alloys, solute atoms are observed in higher concentrations in a volume surrounding dislocations, commonly referred to as a Cottrell Atmosphere^20^. Nearby solute atoms are attracted by the strain field associated with the dislocation from a region that we describe here as a ‘capture zone'. The size of this zone varies between elements since it depends on the lattice diffusion rate, which is affected by temperature, the relative sizes of the solute and matrix atoms and the bonding type^21^. The solute may follow dislocations as they move through the lattice, but only up to a critical velocity. Above this velocity, the dislocation and solute become detached^3^. Additional substantial element mobility may occur through pipe diffusion, the process of relatively rapid diffusion of atoms along dislocation cores^22^,^23^. Although in metals pipe diffusion has been demonstrated to occur along single dislocations^24^,^25^, evidence for pipe diffusion occurring in minerals is so far only indirect^17^,^18^,^19^,^26^.

Here we present an atomic-scale study of a ∼2.5-Ga-old zircon grain using a range of high-end microanalytical techniques including atom probe tomography (APT)^27^, high-resolution electron backscatter diffraction (EBSD), transmission Kikuchi diffraction (TKD)^28^ and sensitive high-resolution ion microprobe (SHRIMP). Our work shows that deformation induced dislocation movement and dislocation structures can significantly influence element mobility and redistribution in a crystalline material. As such, when interpreting local elemental and isotopic variations in both deforming and deformed crystalline materials, a thorough characterization of deformation-related dislocation structures is essential.

## Results

### Sample selection and general sample characteristics

The zircon selected for this study needed to satisfy three main criteria: (i) a sufficiently high U and Pb content to allow reliable, statistically robust detection of radiogenic isotopes using APT, (ii) a known crystal–plastic deformation history to enable an understanding of the link between deformation and element mobility and (iii) a sufficiently long time after the main deformation event(s) to allow an assessment of the importance of pre-existing deformation structures on trace element/isotope mobility in a static regime. The zircon chosen for this study originates from a garnet-orthopyroxene-sapphirine gneiss from the Archean Napier Complex, Antarctica^29^ (Supplementary Note 1). Previous studies show that zircons from this particular area meet the criteria outlined above. Napier complex zircons are generally old (>2 Ga), and have a sufficiently high Pb and U content^30^,^31^,^32^. Deformation occurred in the early history of the zircons at high temperatures ensuring crystal–plastic deformation features with little or no later plastic deformation^29^,^33^,^34^.

**Figure 1:**
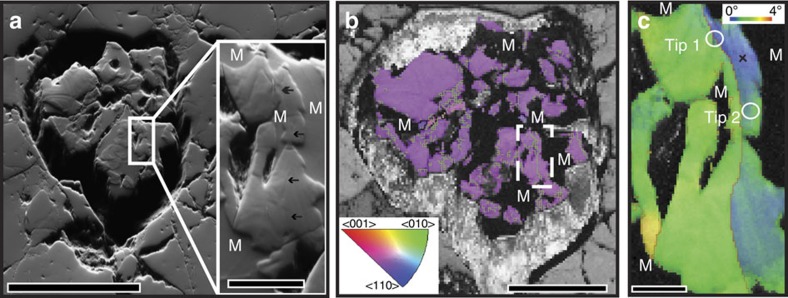
Characteristics of the analysed zircon grain. Metamict domains are marked as M. (**a**) Forescatter electron image; metamict domains are homogeneous, topographic lows; crystalline areas show a complex zonation pattern; scale bar, 100 μm. Inset depicts the analysis area shown in **c**; black arrows point to a low-angle boundary; scale bar, 10 μm. (**b**) Colour-coded orientation EBSD map. Orientation data for the zircon are superimposed on a pattern quality map; low-angle boundaries (>0.8°) are marked in green; white rectangle depicts region of interest shown in **c**; scale bar, 50 μm. (**c**) EBSD relative misorientation map superimposed on a pattern quality map; red lines mark low-angle boundaries (>0.7°); black cross marks the reference point for the relative misorientation colour coding; note location of APT tips 1 and 2; scale bar, 5 μm.

The analysed grain displays a sub-euhedral shape. EBSD analysis reveals complex-shaped metamict domains, characterized by a complete lack of any diffraction patterns and revealed in forescatter electron images as featureless, topographic lows (Fig. 1). EBSD-based orientation data show evidence for crystal–plastic deformation. On this basis, a single well-defined, low-angle boundary with a misorientation of 1–1.5° extending ∼25 μm between two adjacent metamict domains was selected for further analysis (Fig. 1). Detailed misorientation analysis of this boundary shows a rotation about one <011> axis consistent with a tilt boundary oriented close to perpendicular to the sample surface (Supplementary Fig. 1). The surrounding zircon lattice shows evidence of minor crystal plastic deformation with distributed lattice bending accumulating to 1–2° about either <111> or <011> (Fig. 1b and Supplementary Fig. 1). Two needle-shaped specimens or ‘tips' were prepared for atomic-scale analysis with APT from the regions indicated in Fig. 1c: one across the low-angle boundary (APT tip 1) and one less than 1 μm to the side (APT tip 2).

### Atomic-scale analysis

**Figure 2:**
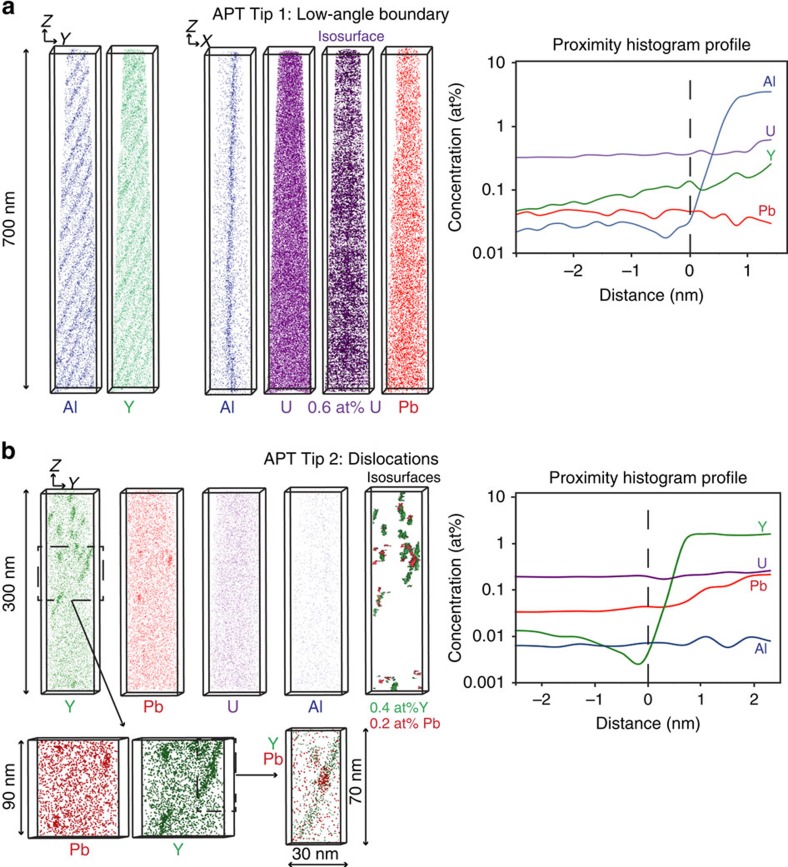
Atomprobe Tomography results. (**a**) APT tip 1; Al, Y, U and Pb atom maps; note different orientation of maps, see Fig. 1c for tip location. (Upper right) Proximity histogram profile based on 0.1 at% Al isosurface in the vicinity of the low-angle boundary. For compositional data, see Table 1. (**b**) APT tip 2; see Fig. 1c for tip location: Al, Y, U and Pb atom maps and isosurfaces of 0.4at% Y and 0.2at% Pb. (Lower left) Enlarged views around the dislocations. (Lower middle) Close-up of a single dislocation; note Pb cluster within the Y-enriched dislocation. (Upper right) Proximity histogram profile in the vicinity of a dislocation. For compositional data see Table 2.

The dislocation array in tip 1 is immediately visible in the APT reconstruction (Fig. 2a and Supplementary Movie 1). The individual dislocations are highlighted by a very high concentration of Al (>2 at. %) and increased concentrations of Y and U (both ∼0.1–0.2 at. %). The U concentration in the dislocation array is ∼3x that in the matrix (compared with 17x and 3,000x for Y and Al, respectively; Table 1). The Pb concentration in the dislocation array is below detection levels (<260 p.p.m.a.) but the proximity histogram shows that there is no increase in the concentration of Pb in the dislocation array relative to the matrix (∼50 p.p.m.a.; Fig. 2a). The dislocation array is oriented parallel to the tip (Fig. 2a and Supplementary Figs 1 and 2) and the individual dislocation spacing is ∼50 nm. Tip 2 shows a very different structure (Fig. 2b and Supplementary Movie 2). In the uppermost 150 nm of the data set, numerous clusters are present that are enriched in both Pb (1800, p.p.m.a., 25x matrix) and Y (17,420 p.p.m.a., >1,700x matrix; Table 2). The elongated morphology of these clusters, their presence in only one part of the volume, and the correlation of this region with a zone of lattice distortion measured by TKD analysis (Supplementary Fig. 2) suggest that these are partially decorated dislocations. There is minor enrichment of U in these dislocations, but no enrichment of Al (Table 2). Tip 2 exhibits additional enrichment of Yb and P in the dislocations, with 3,740 p.p.m.a. (not detected in the matrix) and 9,050 p.p.m.a. (10x matrix), respectively (Table 2).

### Ion microprobe analysis

**Figure 3:**
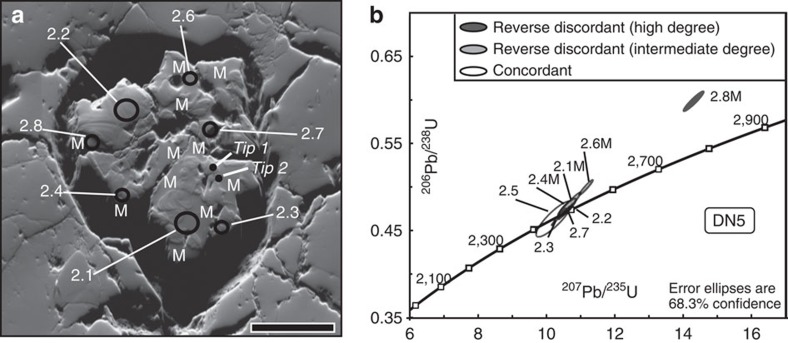
Details on the SHRIMP data from analysed zircon. (**a**) Forescatter electron image of the zircon grain, showing metamict areas (M), which are homogeneous and topographic lows, SHRIMP analysis spots (black circles) and locations for APT tips 1 and 2 (black dots); scale bar, 50 μm; refer to Table 3 for results. (**b**) Conventional Wetherill concordia plot for SHRIMP zircon analyses; spot numbers correspond to those given in **a**; reverse discordant and concordant analyses are grey scale coded.

SHRIMP-based isotopic analyses show higher U and Pb concentrations and a greater degree of reverse discordance (that is, a relative enrichment in Pb over U) in metamict domains than in crystalline regions (Table 3 and Fig. 3). Because of the size and depth of the analysed volume, signals may originate from variable mixtures of metamict and crystalline domains. The ^207^Pb/^206^Pb ages for 7 of the 8 analyses are 2,470±10 Ma (Table 3 and Fig. 3).

## Discussion

**Figure 4:**
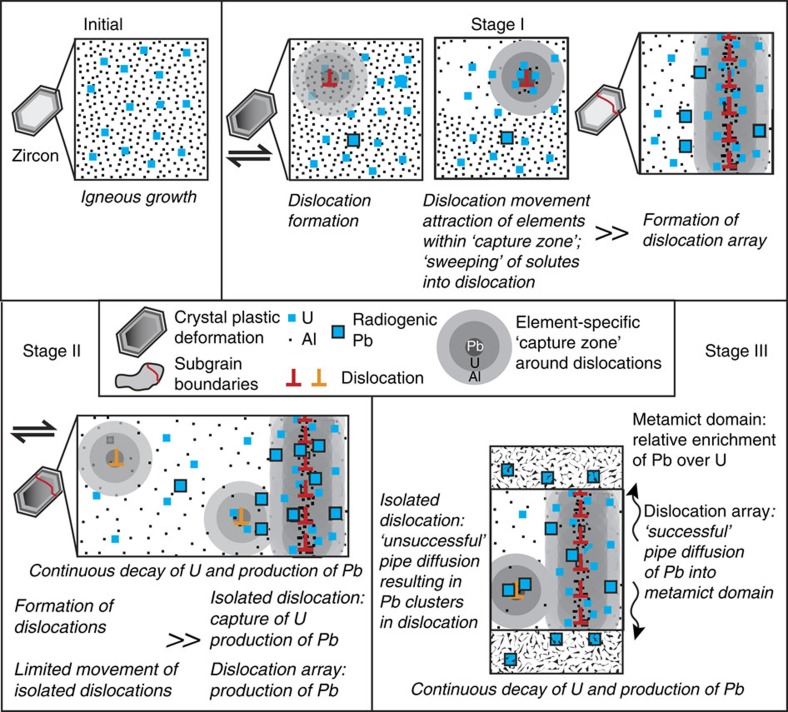
Schematic diagram showing the link between element redistribution and dislocations. This diagram illustrates the proposed stages of dislocation formation and movement and associated trace element mobility within this zircon grain. Only the trace elements U, Pb and Al are considered, with active processes shown in italics. See text for further details.

The correlation between the deformation microstructures and the atomic-scale distribution of trace elements in this zircon allows us to determine the mechanisms of trace element movement and segregation in a deformed mineral. The sample analysed is characterized by a series of high temperature deformation events at common geological strain rates (∼10^−11^–10^−16^ s^−1^) with peak temperatures exceeding 980 °C (refs 29, 33, 34, 35). The exact timing of these events has been the subject of much discussion, but the general consensus is that there was high temperature formation and deformation of zircons at around 2.55–2.45 Ga, followed by some weaker, lower temperature (∼500–600 °C) retrograde deformation. We believe that the dislocation array in tip 1 was formed during the early stages of deformation at relatively high temperatures (>800 °C) enabling dislocation movement through the lattice by both glide and climb. High temperatures result in higher lattice diffusion rates within the crystal lattice, which will enlarge the capture zone, although the bonding of trace elements to the dislocation core may be weakened. At the same time, at high temperatures, the elastic strain may be dissipated by increased dislocation movement. The higher level of dislocation movement, together with an enlarged capture zone (outweighing the effect of a possible weak element-dislocation bond), means that much of the zircon lattice volume was at some point in time in a dislocation capture zone, resulting in large volumes becoming solute depleted (Fig. 4, Stage I). At the same time, the dislocations themselves become enriched (Fig. 4, Stage I) as at the geologically slow strain rates solutes remain captured.

The absence of detectable Pb in the dislocation array in tip 1 is striking. However, the presence of a relatively high amount of U in this array, presumably swept into the array during its formation, would necessitate the presence of a significant amount of radiogenic Pb (Fig. 4, Stage II). Given the age of this zircon and the concentration of U in the array, we would expect ∼550 ppma of Pb to be present in the array. Pb must diffuse along the dislocation array into the structural sink at either end—namely the porous, metamict domains (Fig. 1). In effect, Pb is ‘successfully' lost from the dislocation array (Fig. 4, Stage III). SHRIMP analyses of such metamict domains do indeed show a reverse discordance with higher than expected Pb levels (Fig. 3, Table 3). This process must be ongoing throughout the history of this sample, even after the deformation event and at lower temperatures. This is unequivocal evidence for pipe diffusion along a dislocation array in zircon, resulting in relatively fast and continuous redistribution of Pb over >10 μm.

Reverse discordance has been the subject of a number of studies^13^,^14^,^15^,^36^,^37^ and is generally observed in high-U zircons (above a threshold of ∼2,500 p.p.m. U). The phenomenon has been attributed to possible matrix effects, causing increased relative sputtering of Pb from high-U, metamict regions and resulting in a 1–3% increase in ^206^Pb/^238^U ages for every 1,000 p.p.m. U (ref. 15). However, in the zircon analysed here, the relationship between U content and reverse discordancy is not simple: several points show a degree of reverse discordance despite having U concentrations below 2,000 p.p.m. (spots 2.2 and 2.4, Table 3), whereas the highest measured reverse discordancy (21%, spot 2.8, Table 3) is from a location with 3,102 p.p.m. U, only just above the threshold for which reverse discordancy is normally attributable to matrix effects. Different to analyses exhibiting some degree of reverse discordance, we interpret that the chemical signal of spot 2.8 represents a domain that is completely metamict. Although we cannot rule out some matrix effects in high-U, metamict zones, the complex relationship between U content and reverse discordancy in this zircon is further evidence for an additional process of Pb-enrichment—namely the pipe diffusion of Pb along dislocation arrays into adjacent metamict zones.

In tip 2, the dislocations have not formed into well-ordered arrays indicating little, high-temperature recovery took place. The lack of Al in both the matrix and dislocations in tip 2 and its proximity to the low-angle boundary in tip 1 suggests that the dislocations forming the array in tip 1 had already passed through this volume, collecting the majority of the solute atoms, especially Al, and to a lesser extent U and Pb. As a consequence, there is only a slight enrichment of ‘swept' U in the dislocations of tip 2 (Fig. 4, Stage II). In the dislocations, Pb concentrations are too high to be explained by radioactive decay of U alone. We believe that the Pb clustering in particular areas along the dislocation is further evidence for its localized redistribution through pipe diffusion along dislocation cores (Fig. 4, Stage III). However, in contrast to tip 1, the lack of a physically continuous connection to a sink leads to ‘unsuccessful' pipe diffusion: there is no loss of Pb from the dislocation (Fig. 4, Stage III). Consequently, at the micrometre scale, no element redistribution takes place. Radiation damage may enhance this pipe diffusion/clustering behaviour. The phenomena we observe here is quite similar to the segregation and clustering behaviour around dislocations in metals^38^.

Even though, in the field of materials science, pipe diffusion plays a very important role in governing the properties of advanced materials, affecting nucleation, corrosion, creep and dynamic strain-ageng^39^,^40^, it has proven to be extremely hard to demonstrate or measure its effects experimentally, as processes are too fast to allow a time-resolved study. Our study in zircon provides a unique opportunity to study this phenomenon with a control on timing, as the continuous production of elements through radioactive decay is time dependent. Consequently, this study is as close as possible to an ‘*in situ*' study allowing the construction of a robust model with regard to ongoing processes through time. Therefore, we are able to provide unprecedented evidence of pipe diffusion along a dislocation array, a process of key interest to the materials community.

Our results demonstrate the importance of deformation processes and microstructures on the localized trace element concentrations and continuous redistribution from the nanometre to micrometre scale in the mineral zircon. Dislocation movement through the zircon lattice can effectively sweep up and concentrate solute atoms at geological strain rates. Dislocation arrays can act as fast pathways for the diffusion of incompatible elements such as Pb across distances of >10 μm if they are connected to a chemical or structural sink. Hence, nominally immobile elements can become locally extremely mobile. Not only does our study confirm recent speculation that an understanding of the deformation microstructures within zircon grains is a necessity for subsequent, robust geochronological analyses but it also sheds light on potential pit-falls when utilizing element concentrations and ratios for geological studies. Our results have far-reaching implications for the interpretation of local elemental variations in not only deformed minerals but also a range of engineering materials.

## Methods

### Scanning electron microscopy

A thin section from the analysed sample DN5 was mechanically polished down to 1 μm, and subsequently mechano-chemically polished to allow EBSD analysis. The thin section was coated with ∼5–10 nm carbon, and then candidate zircon grains were detected using a combination of backscattered electron imaging and energy dispersive X-ray spectroscopy (EDS) using a Zeiss EVO scanning electron microscope (SEM) and an Oxford Instruments INCA EDS system (Australian Centre for Microscopy and Microanalysis, University of Sydney, Australia). The zircon presented in this study was subsequently analysed in a Zeiss Ultra Plus field emission gun SEM using an Oxford Instruments AZtec integrated EBSD/EDS system (Australian Centre for Microscopy and Microanalysis, University of Sydney, Australia). A low-resolution EBSD-EDS map was collected using a step size of 1 μm, indexing the orientations of the zircon grain and the surrounding phases—quartz, orthoclase and antiperthitic feldspar. EBSD data were processed using CHANNEL5 software in order to produce orientation, boundary and relative orientation maps. A region of interest, including low-angle boundaries not associated with brittle fractures, was selected and was further analysed using EBSD at higher resolution, with a step size of 200 nm. EBSD data were cleaned using a single isolated pixel removal process, and then with one pass to remove unindexed pixels with five or more indexed neighbours. Metamict regions exhibited no diffraction because of radiation damage to the crystal lattice; hence, these regions could not be indexed using EBSD and appear black in pattern quality maps.

### Sample preparation for APT and TKD

Rectangular regions of interest measuring ∼3 × 2 μm were selected on the basis of the EBSD data, and cut free using a Zeiss Auriga focused ion beam SEM (Australian Centre for Microscopy and Microanalysis, University of Sydney, Australia). The rectangular prisms were lifted out using a Kleindiek micromanipulator system and welded onto electropolished molybdenum grids using platinum deposition. The samples were then milled to form atom probe tips, 80–120 nm in diameter, with final milling using a low acceleration voltage (10 kV) in order to minimize Ga implantation and damage. The tip long axes were oriented perpendicular to the original polished grain surface.

### TKD analysis

TKD analysis provides orientation information with a higher spatial resolution than EBSD^28^, allowing characterization of plastic deformation within the tip and subsequent correlation with atom probe data. The TKD signal arises from the lower surface of the tip, so that a sub-horizontal boundary orientation (such as in APT tip 1) would only be apparent where the boundary intersects the lower surface and at the very tip end (see Supplementary Fig. 3 for details). As for the EBSD measurements, TKD analyses were performed using a Zeiss Ultra Plus field emission gun SEM equipped with an Oxford Instruments AZtec EDS/EBSD system (Australian Centre for Microscopy and Microanalysis, University of Sydney, Australia). Each APT tip was mapped using a step size of 10 nm, an accelerating voltage of 30 kV and a probe current of 10–20 nA. Orientation maps were processed in the same way as for EBSD data.

### APT measurements and analysis

APT measurements were conducted on a Cameca LEAP 4000X Si atom probe equipped with a picosecond-pulse ultraviolet laser (Australian Centre for Microscopy and Microanalysis, University of Sydney, Australia). In laser-pulsed APT, a sharp needle with a typical diameter of ∼100 nm is placed under an intense field produced by a DC voltage of up to 10 kV, in high vacuum and cryogenic temperature (between 20 and 100 °K). Ultra-short laser pulses are then used to field evaporate atoms from the specimen surface. A position-sensitive detector detects the ions and their mass-to-charge ratios are recorded by time-of-flight spectroscopy, synchronized with the laser pulse.

In our study, a 355-nm wavelength laser with a pulse energy of 400 pJ and 250 kHz pulse frequency was used to field evaporate both samples at 50 °K. A total of 68 million atoms in tip 1 and 23 million atoms in tip 2 were detected. The mass-resolving power was around 750 full-width at half-maximum for both samples (measured on O^+^ peak; Supplementary Fig. 3).

The minimum detection limit is dependent on the number of atoms considered and the position of the peaks within the mass spectrum and is approximately 10 p.p.m.a. The spatial resolution is generally below 0.3 nm in *X*, *Y* and *Z*.

Molecular species and isobaric interferences are quite common for high-resistivity materials such as zircon and could lead to inaccuracies in compositional measurements^27^. In the case of the two data sets from this study, the molecular species containing the major elements (Zr, Si, O) were easily identified and discriminated using isotopic ratios. No significant isobaric interference was encountered. Al and Y, important trace elements in our study, evaporated principally as single ions without isobaric interferences from other ions. As for radiogenic Pb, there is an isobaric interference between ^208^Pb^2+^ and ^104^Si_2_O_3_^+^ and as a result only ^206^Pb^2+^ and ^207^Pb^2+^ were used to quantify Pb. A deconvolution of the peak was impossible because of the non-radiogenic character of Pb limiting the use of isotopic ratio. The mass spectra obtained for the two data sets in this study were high quality, with a very good mass resolving power (750 full-width at half-maximum for O^+^), limited hydrides and only a minor thermal tail behind peaks. Our results are in excellent agreement with the study of Hadean and Archean zircons from Valley *et al*., where the mass spectrum full analysis is detailed, providing a benchmark for APT analysis of zircons^41^.

### SHRIMP analysis

Additional high-resolution chemical analyses on the zircon grain were carried out using a SHRIMP (Australian National University, Canberra, Australia). Two spot sizes (8 and 15 μm diameter) were used (for locations see Fig. 3a). Uncertainties given for individual U-Pb analyses (ratios and ages) are at the 1σ level, however, uncertainties in the calculated weighted mean ages are reported as 95% confidence limits and include the uncertainties in the standard calibrations where appropriate. The standard zircon SL13 (U=238 p.p.m.)^41^ was used for the reference value of U and Th concentrations in zircon. Pb/U ratios were corrected for instrumental interelement fractionation using the ratios measured on the standard zircon Temora 2 (416.8±1.3 Ma)^42^. Common Pb corrections were based on the measured ^204^Pb and the relevant common Pb compositions from the Stacey and Kramers model^43^. Data reduction and processing were conducted using the Excel Macros SQUID 2 and ISOPLOT^44^,^45^.

## Additional information

**How to cite this article:** Piazolo, S. *et al*. Deformation-induced trace element redistribution in zircon revealed using atom probe tomography. *Nat. Commun.* 7:10490 doi: 10.1038/ncomms10490 (2016).

## Supplementary Material

Supplementary Information Supplementary Figures 1-3, Supplementary Note 1 and Supplementary References

Supplementary Movie 1 Animation showing the 3D distribution of Al, Y, U and Pb atoms in a dislocation array, APT tip 1.

Supplementary Movie 2 Animation showing the 3D distribution of Y and Pb atoms in a partially decorated dislocation; APT tip 2.

## Figures and Tables

**Table 1:** Chemical analysis from the APT tips: APT tip 1 for the low-angle boundary (where Al >0.1 at%) and matrix (where Al <200 p.p.m.a.).

*APT tip 1*	*Matrix (∼ 68 Mio. atoms)*	*Low-angle boundary* *(∼120,000 atoms)*
O (at%)	68.8±0.02	67.3±0.3
Zr (at%)	15.8±0.01	14.2±0.2
Si (at%)	14.9±0.01	15.3±0.2
Al (p.p.m.a.)	8±3	24,270±520
Y (p.p.m.a.)	110±3	1,830±140
Pb (p.p.m.a.)	50±4	ND*
U (p.p.m.a.)	357±3	965±100
Hf (p.p.m.a.)	1,765±5	1,910±140
Mg (p.p.m.a.)	18±3	ND
P (p.p.m.a.)	378±4	180±40
Li (p.p.m.a.)	67±3	ND
Be (p.p.m.a.)	25±3	ND

APT, atom probe tomography; ND, not detected.

^*^Detection limit over 120,000 atoms: 260  p.p.m.a.

**Table 2:** Chemical analysis from the APT tips: APT tip 2 for dislocations (where Y >0.1 at%) and the matrix (where Y <400 p.p.m.a.).

*APT tip 2*	*Matrix (∼23 Mio. atoms)*	*Dislocations (∼198,000 atoms)*
O (at%)	66.5±0.02	66.6±0.3
Zr (at%)	16.8±0.01	15.5±0.2
Si (at%)	15.8±0.01	13.9±0.2
Al (p.p.m.a.)	8±3	ND
Y (p.p.m.a.)	10±3	17,420±270
Pb (p.p.m.a.)	71±4	1,800±100
U (p.p.m.a.)	346±3	420±50
Hf (p.p.m.a.)	2,650±7	2,200±140
Yb (p.p.m.a.)	ND	3,740±150
Mg (p.p.m.a.)	10±3	ND
P (p.p.m.a.)	900±5	9,050±200
Ta (p.p.m.a.)	14±3	120±30
Li (p.p.m.a.)	96±3	ND
Be (p.p.m.a.)	15±3	ND

APT, atom probe tomography; ND, not detected.

**Table 3:** Summary of SHRIMP U-Pb zircon data.

Grain. spot	% ^206^Pb_c_	p.p.m. U	p.p.m. Th	^232^Th /^238^U	±%	(†) p.p.m. ^206^Pb^*^	(†) ^206^Pb /^238^U Age		(†) ^207^Pb /^206^Pb Age		% Discordant	(†) ^207^Pb^*^/^206^Pb^*^	±%	(†) ^207^Pb^*^/^235^U	±%	(†) ^206^Pb^*^/^238^U	±%	Err corr
2.1	0.01	5,209	1,214	0.24	2.34	2,147	2,527	±39	2,471	±9	−3	0.161	0.6	10.7	1.9	0.48	1.9	1.0
2.2	0.01	1,615	102	0.07	0.46	661	2,511	±30	2,472	±14	−2	0.162	0.8	10.6	1.6	0.48	1.4	0.9
2.3	0.02	2,018	331	0.170	2.0	621	2,465	±28	2,472	±3	+0	0.16156	0.17	10.38	1.4	0.466	1.4	0.99
2.4	0.03	1,900	310	0.169	2.2	597	2,556	±25	2,481	±12	−4	0.16238	0.68	10.89	1.4	0.487	1.2	0.87
2.6	0.01	4,209	1,019	0.250	1.4	1,211	2,613	±24	2,478	±6	−7	0.16209	0.36	11.17	1.2	0.500	1.1	0.95
2.7	0.06	2,712	467	0.178	2.4	844	2,486	±22	2,483	±3	−0	0.16262	0.17	10.55	1.1	0.471	1.0	0.99
2.8	0.15	3,102	847	0.282	0.2	1,138	3,024	±31	2,592	±8	−21	0.17357	0.46	14.32	1.4	0.598	1.3	0.94

SHRIMP, sensitive high-resolution ion microprobe.

Errors are 1-sigma; Pb_c_ and Pb* indicate the common and radiogenic portions, respectively.

Error in standard calibration was 0.33% (not included in above errors but required when comparing data from different mounts).

†Common Pb corrected using measured ^204^Pb.
