# Supplementary material for: Deformation-induced trace element redistribution in zircon revealed using atom probe tomography
Source: Nat Commun. 2016 Feb 12;7:10490. doi: 10.1038/ncomms10490 (PMC4754339; doi:10.1038/ncomms10490)
Supplement: Supplementary Information — Supplementary Figures 1-3, Supplementary Note 1 and Supplementary References [file ncomms10490-s1.pdf]

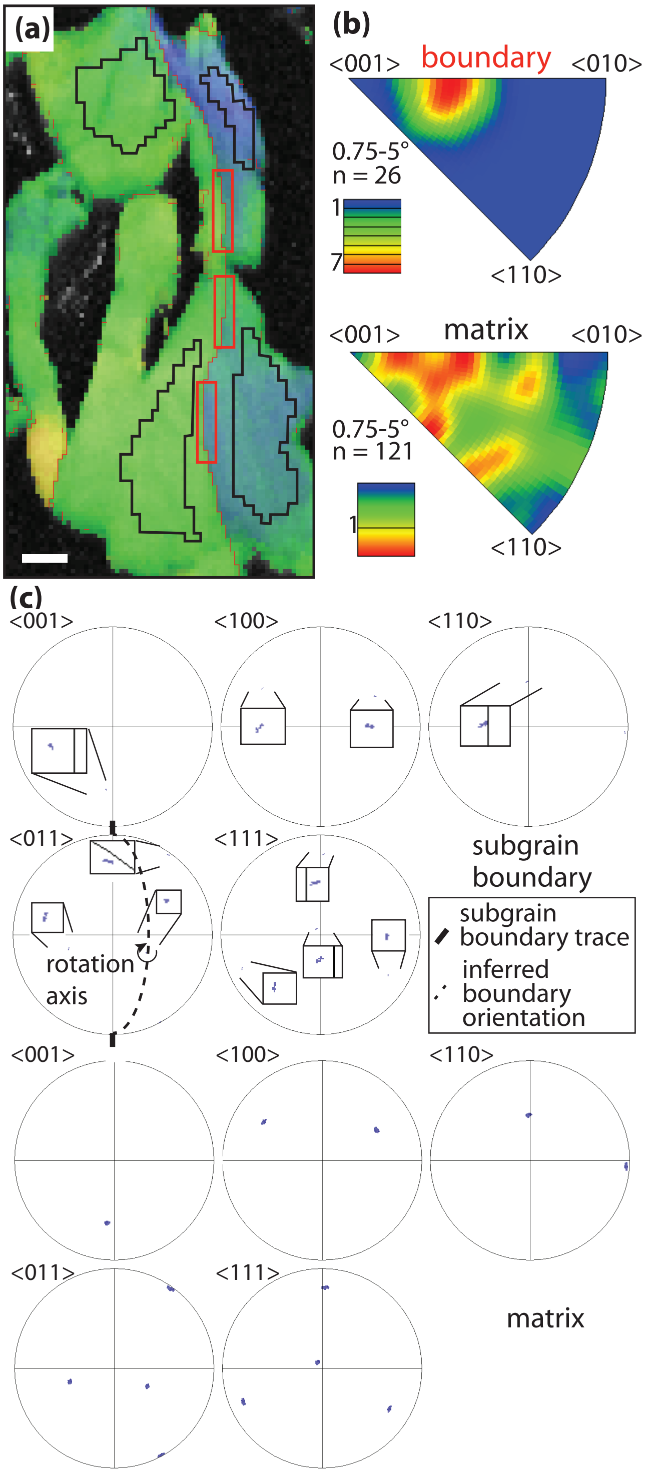

Supplementary Figure 1: Additional orientation data from the area presented in Fig. 1c

(a) EBSD map showing the location of two orientation datasets, representing data from the subgrain boundary (red) and in the area of isolated dislocations (black); scale bar represents 2 μm. (b) Misorientation axes for both areas shown in an inverse pole figure; note the cluster of misorientation axes with rotation around <011> for the subgrain boundary data set, which is significantly weaker in the matrix. (c) Pole figure of the same two areas; a clear rotation axis is only observed for the subgrain boundary dataset; the boundary trace (solid line) and inferred boundary plane (stippled) are also shown. The fact that the rotation axis lies in the boundary plane is consistent with a tilt boundary.

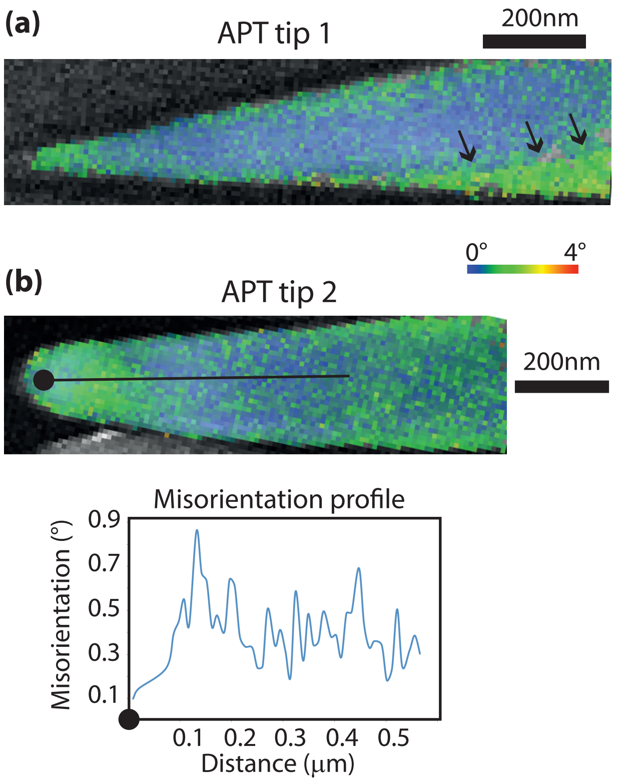

Supplementary Figure 2:
Transmission Kikuchi Diffraction results of APT tips.

Shown here is the local change in crystallographic orientation of the two analysed tips. Data was acquired after annular milling and before insertion of the tips in the LEAP. Note that only a part of these samples were field evaporated in the LEAP (about a 700 nm length for tip 1 and 350 nm for tip 2). **(a)** APT tip 1; note the low angle boundary in the lower part of the tip (black arrow); step size is 10 nm. **(b)** APT tip 2: note the relative change in orientation along a profile across the first

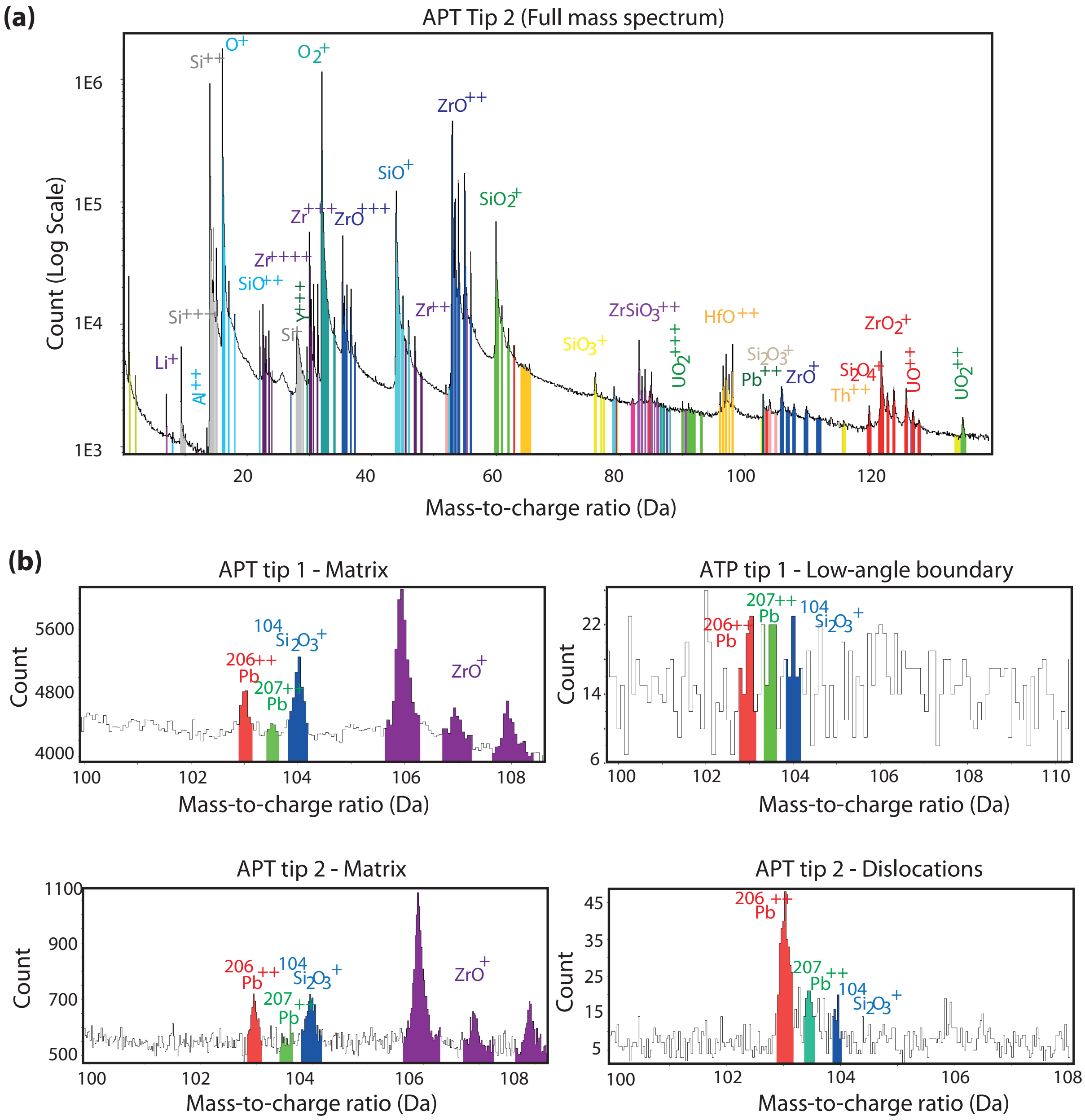

Supplementary Figure 3: APT mass spectra for the two tips presented in this study.

(a) APT tip 1; full mass spectrum (logarithmic scale) from 1 to 140 Da; note many elements are detected as ionic molecules and have multiple charges. **(b)** Enlarged area of the mass spectra (linear scale) for both tips from 100 Da to 108 Da, showing 206Pb++, 207Pb++, 104Si2O3+ and ZrO+. Note that no lead is observed in the low-angle boundary (below detection limit) (APT tip 1) and a high lead content is detected in the isolated dislocations (APT tip 2).

Supplementary Note 1: Geological background and sample description

The sample DN5 is from a locality on the lower slopes of Mount Pardoe (67°08’35.6”S, 50°13’27.6”E) which is situated on the south-eastern side of Amundsen Bay in the Napier Complex (66°-68°S, 48°-57° E), Antarctica. The Napier Complex is an Archean granulite facies terrain belonging to the East Antarctic Precambrian Shield and hosts ultrahigh temperature (UHT) metamorphic rocks at a regional scale (1, 2, 3). A general description of the region is provided by (1, 2) and in reviews by (4) and (5). Peak metamorphic conditions across the Napier Complex are >1000 ^{o}C at pressures from ~11 kbar in the southern part to ~6 kbar in the northern part (3, 6, 7, 8). Mount Pardoe itself belongs to the central-western part of Napier Complex, within the region of sapphirine-quartz stability and close to Tonagh Island, where pressures of 8-9 kbar have been estimated for the metamorphic peak (3, 4). In this part of the Napier Complex large scale near-isoclinal reclined to recumbent D2 folds fold pre-existing high grade foliations developed during the first deformation event (D1). Such D2 refolding and associated layer-parallel flattening and megaboudinage is observed on metre to 100 metre fold amplitude scales at Mt Pardoe in the vicinity of the DN5 locality. The DN5 locality features a 20 metre thick sequence of pale gneisses including: garnet-quartz-feldspar-bearing massive gneiss; layered quartzofeldspathic felsic gneiss; garnet-rich pods containing sapphirine, orthopyroxene and feldspar with a secondary garnet, and boudinaged garnetite pods and layers. The pale gneisses and associated garnetite pods are considered to have formed from the metamorphism of sedimentary protoliths, based on the range in inferred bulk-rock compositions in the pale gneiss sequence and the geochemistry of comparable paragneisses from the Napier Complex (1, 2). Broadly concordant pods of very coarse grained garnet-orthopyroxene restitic material occurs with and surrounds irregular leucosomes of quartz-feldspar-rutile interpreted to be syn-D1/D2. The restitic pods may

contain detrital zircons inherited from the sedimentary protoliths to the gneisses and zircons formed during the crystallisation of the irregular leucosomes.

The sample studied represents such a silica-deficient boudinaged horizon, consisting of garnet, sapphirine (Spr), orthopyroxene (Opx) and feldspar. In thin section, different orthopyroxene (Opx) textures are seen. Opx occurs either as coarse blasts that enclose subhedral and skeletal garnet, or as an Opx+Spr assemblage where Opx is typically xenomorphic on subhedral coarse Spr. Up to 2 cm large garnet blasts are observed. These may contain inclusions and inclusion arrays of: Spr, Opx, biotite, antiperthitic plagioclase, rutile, and in rare cases sillimanite. A second generation of garnet is seen associated with Spr+Opx, next to Spr adjacent to feldspar, and in places it is also associated with minor sillimanite. Both antiperthitic and perthitic (mesoperthite) feldspars are present, though in essentially different domains. Feldspars are invariably deformed and exhibit deformation lamella. Within coarse grains wavy, curved and dispersed extinction is observed. Grain boundaries, originally broadly lobate to subhedral, are sutured to serrated on finer scales, modified by recrystallisation. Seams of similarly finer-grained neoblastic feldspar occur within and between grains and are present at garnet and feldspar contacts where late biotite and sillimanite also occurs. The analysed zircon grain is embedded in quartz, orthoclase and antiperthitic feldspar. The surrounding phases all exhibit irregular boundaries and undulose extinction.

Supplementary References

1. Sheraton, J. W., Tingey, R. J., Black, L. P., Offe, L. A. & Ellis, D. J. Geology of Enderby Land and Kemp Land Antarctica. Aus. Bureau Min. Res. Bull. **223**, 1-51 (1987).

2. Sheraton, J. W., Offe, L. A., Tingey, R. J. & Ellis, D. J. Enderby Land, Antarctica — an unusual Precambrian high-grade metamorphic terrain. Journal of the Geological Society of Australia **27**, 1–18 (1980).
3. Harley, S. L. Garnet–orthopyroxene bearing granulites from Enderby Land, Antarctica: metamorphic pressure–temperature–time evolution of the Archean Napier Complex. Journal of Petrology **26**, 819-856 (1985).
4. Harley, S. L. & Hensen B. J. Archaean and Proterozoic high-grade terranes of East Antarctica (40–80 E): a case study of diversity in granulite facies metamorphism. High-temperature metamorphism and crustal anatexis. Springer Netherlands, pp. 320-370 (1990).
5. Harley, S. L. Ultrahigh temperature granulite metamorphism (1050 °C, 12 kbar) and decompression in garnet (Mg70)- orthopyroxene–sillimanite gneisses from Rauer Group, East Antarctica. Journal of Metamorphic Geology **16**, 541–562 (1998).
6. Harley, S. L. & Motoyoshi, Y. Al zoning in orthopyroxene in a sapphirine quartzite: evidence for N1120 °C UHT metamorphism in the Napier Complex, Antarctica, and implications for the entropy of sapphirine. Contributions to Mineralogy and Petrology **138**, 293-307 (2000).
7. Hokada, T. Feldspar thermometry in ultrahigh-temperature metamorphic rocks: evidence of crustal metamorphism attaining ~1100 °C in the Archean Napier Complex, East Antarctica. American Mineralogist **86**, 932–938 (2001).
8. Lund, M. D., Piazolo, S. & Harley, S. L. Microtextures and deformation mechanisms in deformed felsic high grade granulites: Insights from EBSD analysis. Tectonophysics **427**, 133-151 (2006).
